# Cervicovaginal Fluid Acetate: A Metabolite Marker of Preterm Birth in Symptomatic Pregnant Women

**DOI:** 10.3389/fmed.2016.00048

**Published:** 2016-10-10

**Authors:** Emmanuel Amabebe, Steven Reynolds, Victoria Stern, Graham Stafford, Martyn Paley, Dilly O. C. Anumba

**Affiliations:** ^1^Academic Unit of Reproductive and Developmental Medicine, University of Sheffield, Sheffield, UK; ^2^Academic Unit of Radiology, University of Sheffield, Sheffield, UK; ^3^Integrated BioSciences, School of Clinical Dentistry, University of Sheffield, Sheffield, UK

**Keywords:** cervicovaginal fluid, microbiota, metabolite, acetate, preterm birth

## Abstract

Changes in vaginal microbiota that is associated with preterm birth (PTB) leave specific metabolite fingerprints that can be detected in the cervicovaginal fluid (CVF) using metabolomics techniques. In this study, we characterize and validate the CVF metabolite profile of pregnant women presenting with symptoms of threatened preterm labor (PTL) by both ^1^H-nuclear magnetic resonance spectroscopy (NMR) and enzyme-based spectrophotometry. We also determine their predictive capacity for PTB, singly, and in combination, with current clinical screening tools – cervicovaginal fetal fibronectin (FFN) and ultrasound cervical length (CL). CVF was obtained by high-vaginal swabs from 82 pregnant women with intact fetal membranes presenting between 24 and 36 weeks gestation with symptoms of threatened, but not established, PTL. Dissolved CVF samples were scanned with a 400 MHz NMR spectrometer. Acetate and other metabolites were identified in the NMR spectrum, integrated for peak area, and normalized to the total spectrum integral. To confirm and validate our observations, acetate concentrations (AceConc) were also determined from a randomly-selected subset of the same samples (*n* = 57), by spectrophotometric absorption of NADH using an acetic acid assay kit. CVF FFN level, transvaginal ultrasound CL, and vaginal pH were also ascertained. Acetate normalized integral and AceConc were significantly higher in the women who delivered preterm compared to their term counterparts (*P* = 0.002 and *P* = 0.006, respectively). The ^1^H-NMR-derived acetate integrals were strongly correlated with the AceConc estimated by spectrophotometry (*r* = 0.69; *P* < 0.0001). Both methods were equally predictive of PTB <37 weeks (acetate integral: AUC = 0.75, 95% CI = 0.60–0.91; AceConc: AUC = 0.74, 95% CI = 0.57–0.90, optimal predictive cutoff of >0.53 g/l), and of delivery within 2 weeks of the index assessment (acetate integral: AUC = 0.77, 95% CI = 0.58–0.96; AceConc: AUC = 0.68, 95% CI = 0.5–0.9). The predictive accuracy of CVF acetate was similar to CL and FFN. The combination of CVF acetate, FFN, and ultrasound CL in a binary logistic regression model improved the prediction of PTB compared to the three markers individually, but CVF acetate offered no predictive improvement over ultrasound CL combined with CVF FFN. Elevated CVF acetate in women with symptoms of PTL appears predictive of preterm delivery, as well as delivery within 2 weeks of presentation. An assay of acetate in CVF may prove of clinical utility for predicting PTB.

## Introduction

Approximately 15 million babies are born prematurely annually. Preterm birth (PTB, birth before 37 weeks of gestation), is a global problem costing health care resources in excess of $26 billion in the USA and £3 billion in the UK annually. It is the commonest cause of infant morbidity and mortality worldwide. About 35% of the world’s 3.1 million neonatal deaths annually are due to complications of PTB. Many surviving preterm babies face a lifetime of disability including cerebral palsy, learning, visual, and respiratory disorders amongst others (1–3). Preventing prematurity remains limited by poor prediction, and current tests to identify women at risk are inadequate.

Although the etiology of PTB is multifactorial, infection and inflammation are associated with most spontaneous preterm deliveries (3). However, the etiological mechanisms and pathogenesis of these observations are unclear. Changes in the vaginal microecology of commensal and potentially pathogenic organisms such as bacterial vaginosis (BV) – a polymicrobial vaginal infection common in reproductive-aged women – may influence the initiation of PTB (3). BV is characterized by a shift (dysbiosis) from the healthy *Lactobacillus*-dominated vaginal microflora to an abnormal microbial environment dominated by mixed anaerobes e.g., *Gardnerella, Fusobacterium, Bacteroides, Prevotella, Mobiluncus, Mycoplasmas*, etc. These anaerobes synthesize metabolic acids such as acetate, butyrate, propionate, and succinate, thereby increasing the vaginal pH and inhibiting chemotaxis of immunocompetent cells (4–6). This, in turn, enhances the proliferation of these infectious organisms, leading to ascending intrauterine infection and the establishment of an NF-κB-coordinated inflammatory state (6) that recruits pro-inflammatory cytokines (e.g., IL-1β, IL-4, IL-6, IL-17, TNF-α, and INF-γ) and chemokines (e.g., IL-8 and RANTES), stimulating the production of matrix metalloproteinases (MMPs) and arachidonic acid metabolites such as prostaglandins (PGs) and hydroxyeicosatetraenoic acids (HETE). The MMPs degrade and digest the cervical extracellular matrix and fetal membranes, while the arachidonic acid metabolites modulate myometrial contractility leading to cervical remodeling, preterm labor (PTL), premature membrane rupture, and ultimately PTB (3, 7–10).

Determination of vaginal microbiota metabolite signatures has provided clinically useful insight into the pathophysiology of ascending genital tract infection and subsequent reproductive outcome (11–13). Metabolic acids (e.g., acetate and succinate) produced in large amounts by female genital microbiota (dominated by mixed anaerobic bacteria and deficient in the protective lactic acid-producing *Lactobacillus* species) have been shown to exhibit deleterious immunomodulatory functions (6). These include increasing the pH of the vaginal ecosystem, inducing the production of pro-inflammatory cytokines, and paralyzing the chemotaxis of neutrophils, monocytes, and other immunocompetent cells (4, 5, 14). These encourage luxuriant growth and proliferation of potentially pathogenic bacteria, ascending genital infection, microbial invasion of the amniotic cavity, and inflammation of the fetal membranes and other gestational tissues. The cumulative effect of this sequence of events during gestation is the release of PGs and MMPs, and consequently cervical remodeling, myometrial contraction, preterm premature rupture of membranes, PTL, and preterm delivery (3, 8, 10, 15–17).

Interestingly, the changes in the vaginal microbiota also leave specific signature metabolite fingerprints that can be detected in the cervicovaginal fluid (CVF) using metabolomics techniques such as ^1^H-nuclear magnetic resonance spectroscopy (NMR) (11, 13). In addition to enhancing our understanding of the pathogenesis of inflammation-induced PTB, it is plausible that CVF metabolite profiling can enable the development of clinical predictive tests for identifying women at risk of PTB. Besides, some of these metabolites have been useful in the diagnosis of BV (12), and more recently, we have demonstrated the predictive potential of CVF acetate (derived by ^1^H-NMR) for PTB especially in symptomatic pregnant women (18). Hence, in this study employing commercial spectrophotometric assay techniques with potential for clinical applicability, we validated metabolite profiles previously determined by ^1^H-NMR spectroscopy in CVF obtained from a large cohort of pregnant women presenting with symptoms suggestive of threatened PTL and determined their prognostic capacity for PTB singly and in conjunction with widely employed clinical assessment methods such as quantitative fetal fibronectin (FFN) and ultrasound cervical length (CL). We hypothesized that, compared to their term-delivered counterparts; symptomatic pregnant women who ultimately delivered prematurely would have significantly different CVF metabolite profiles with predictive accuracies comparable to current clinical tests such as cervicovaginal FFN and ultrasound-derived CL.

## Materials and Methods

### Study Cohort and Sampling

Cervicovaginal fluid was obtained by high-vaginal swabs (Deltalab Eurotubo 300263, Fisher Scientific, UK) from pregnant women presenting to the Triage Delivery Suites of the Jessop Wing Hospital, Sheffield, UK, with symptoms suggestive of threatened, but not established, PTL (i.e., regular uterine contractions at least once every 10 min and cervical dilatation <3 cm), between 24 and 36 weeks gestation (*n* = 82). Women with multiple gestation, symptoms or signs of genitourinary infection, and history of abnormal cervical cytology in the last 3 years, ruptured fetal membranes, and prior vaginal examinations at presentation were excluded from the study. Study participants were recruited between January 2014 and September 2015 and closely monitored until delivery outcome was ascertained.

Vaginal swabs were obtained from participants prior to any vaginal examination or clinical treatment intervention such as therapy with antibiotics, steroids, tocolytics, or any vaginal pessary. The obtained CVF samples were instantly processed or stored at −20°C in preparation for metabolite analyses. At presentation, clinical research staff also ascertained CVF FFN, ultrasound CL, and vaginal pH. The clinical course and delivery outcomes of participants were subsequently ascertained.

These studies were approved by the Yorkshire & Humber (Sheffield) Committee of the UK National Research Ethics Service (REC Number 13/YH/0167).

### ^1^H-NMR Spectroscopy

Cervicovaginal fluid samples were prepared and analyzed by ^1^H-NMR as previously described (18). Dacron swab saturated with CVF obtained from the posterior fornix of the vagina was immediately returned to the sterile transport tube, labeled correctly, and stored briefly at −20°C pending extraction into solution. A physiological extraction solution of 600 μl Phosphate Buffered Saline at pH 7.4 was added to the cut end of the swab saturated with CVF sample in a clean 1.5 ml microfuge tube and vortexed for 5 min. The swab was safely discarded after CVF had been washed off into solution. Following centrifugation (13,000 rpm × 3 min), the supernatant was separated and carefully aspirated into a different clean 1.5 ml microfuge tube and stored at −80°C ready for analysis. Four hundred microliters of each NMR sample comprising of 380 μl of CVF in solution and 20 μl of deuterium oxide (D_2_O) was transferred into a 5 mm NMR sample tube (Norell, Marion, NC, USA), ready for scanning.

^1^H-NMR spectra of CVF samples were acquired at approximately 294 K with a 9.4T (400 MHz) Bruker Avance III MR spectrometer (Bruker BioSpin GmbH, Karlsruhe, Germany), with 5 mm broadband observe probe using a Watergate water suppression pulse sequence (number of scans, NS = 256, relaxation time, D1 = 5 s, acquisition time, AQ = 1 s, sweep width, SW = 20.6 ppm, time domain, TD = 16446). ^1^H-NMR spectra phase and baseline correction as well as data processing was performed using the Bruker TOPSPIN 2.1.6 software.

**Figure 1:**
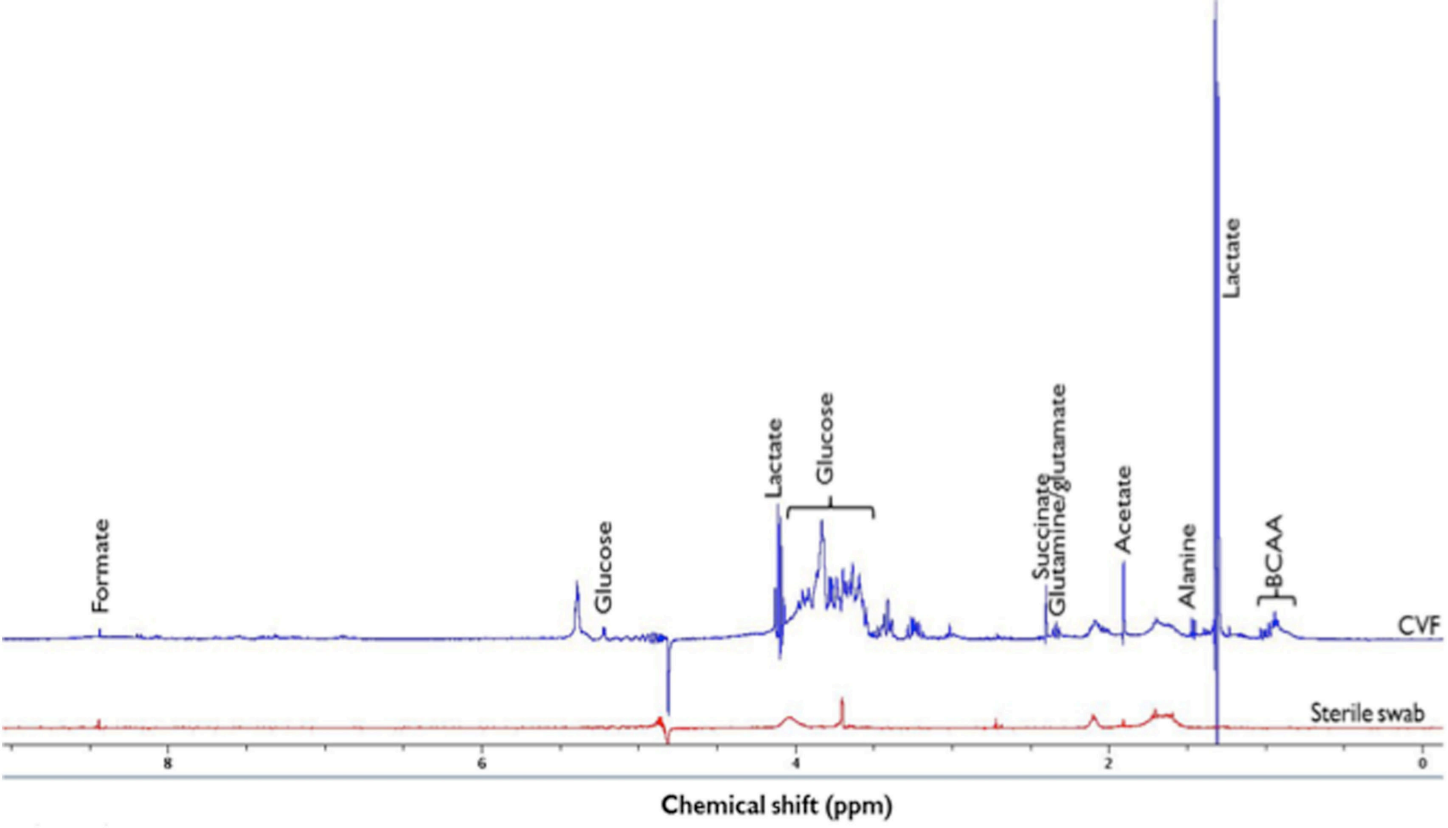
**^1^H-NMR spectrum of identified metabolites in cervicovaginal fluid (CVF) and sterile swab at 400 MHz and 294 K**. Due to the presence of other additional metabolite peaks at the δ = 3.2 ppm and 3.9 ppm region, the glucose signal at δ = 5.2 ppm was integrated and used in subsequent analysis. *BCAA*, Branched chain amino acids (leucine, isoleucine, and valine) (δ = 0.9–1.2 ppm), *ppm*, parts per million.

In order to confirm the structure of the identified metabolites and assign them to the ^1^H-NMR spectral peaks (Figure 1), the following 2-D NMR spectra were acquired ^1^H-^13^C presat-heteronuclear single quantum correlation spectroscopy (HSQC) – NS = 1024, D1 = 1 s, AQ = 0.078 × 0.006 s, SW = 10.0 × 150 ppm, TD = 624 × 180; ^1^H-^13^C presat-heteronuclear multiple bond correlation spectroscopy (HMBC) – NS = 1024, D1 = 1 s, AQ = 0.128 × 0.005 s, SW = 10.0 × 230 ppm, TD = 1024 × 200; ^1^H-^1^H watergate-double quantum filtered correlation spectroscopy (DQFCOSY) – NS = 256, D1 = 0.5 s, AQ = 0.832 × 0.022 s, SW = 9.0 × 9.0 ppm, TD = 6000 × 160; and ^1^H-^1^H presat-clean total correlation spectroscopy (TOCSY) – NS = 16, D1 = 1.5 s, AQ = 0.284 × 0.071 s, SW = 9.0 × 9.0 ppm, TD = 2048 × 512. A representative 2-D ^1^H-^13^C HSQC spectrum is shown in Figure S1 in Supplementary Material. All spectral peaks were referenced to the ^1^H lactate signal at δ = 1.30 ppm. The metabolite peak intensities were then assigned by matching their chemical shifts and multiplicity with previous publications (13, 19), SDBS Spectral Database for Organic Compounds (http://sdbs.db.aist.go.jp/sdbs/cgi-bin/cre_index.cgi), and Chenomx NMR Suite software package (Chenomx Inc., CA, USA, version 7.7). With the same experimental procedures, a sterile (unused) polystyrene Dacron swab was prepared and analyzed as a background control signal.

The identified ^1^H-NMR metabolite signals were integrated for peak area (which is proportional to metabolite concentration) and normalized by dividing each integral by the total spectrum integral (δ = 0.0–10.0 ppm, excluding the residual water signal δ = 4.7–5.0 ppm) to provide a normalized integral (N.I.). This accounted for any differences in CVF concentration or variation in swab sampling.

### CVF Acetate Concentration Measurement

To validate and confirm the data from NMR-derived acetate normalized integrals, predictive accuracy of CVF acetate for PTB and explore potential clinical translation, we determined absolute acetate concentration from a randomly-selected subset of CVF samples (*n* = 57) from the total cohort of participants, by spectrophotometric absorption of NADH using an assay kit for acetic acid (ADP-Glucokinase format, K-ACETGK 08/14, Megazyme, IE). The reaction is a positive reaction indicated by the increase in absorbance as demonstrated by the calibration curve (*R*^2^ = 0.975, detection limit: 1.8 g/l), with its lowest sensitivity limit ~0.1 g/l (*R*^2^ = 0.9997) (Figure S2 in Supplementary Material). The acetic acid GK assay kit is an endpoint type assay specific for acetate. All reagents were prepared, mixed, and stored according to the manufacturer’s instruction. A single reaction mixture containing 3 μl CVF sample in PBS, 200 μl of reagent 1 (distilled water, buffer, and AK/PTA/ADP-GK/G6P-DH), and 20 μl of reagent 2 (NAD + /ATP/D-glucose/CoA/PVP), was assayed for each sample. The reaction time was ~ 5 min at 37°C after which the absorbance of the end product NADH was read at 340 nm. All assays were performed using optical grade 96-well plates on a computer-controlled Infinite M200 microplate reader (TECAN, Chapel Hill) {*wavelength range* = 230–1000 nm; *accuracy*: 0–2 OD: ≤± [1% + 10 mOD; 2–3 OD: ≤±2.5%; *Precision* <0.2% at 260 nm; *Linearity*: *R*^2^ = 0.999 (0–2 OD)]} (see Supplementary Material for more details).

### CVF Fetal Fibronectin, Ultrasound CL, and Vaginal pH

Quantitative CVF FFN levels were determined using the 10Q Rapid FFN analyser (Hologic, MA, USA), vaginal pH using narrow range universal indicator paper (pH-Fix, Machery-Nagel, DE, USA), and CL by transvaginal ultrasonography.

### Data Analysis

All statistical analyses, including receiver operating characteristics (ROC) curve determination were performed using MATLAB (Mathworks, Natick, MA, USA). The Wilcoxon rank-sum test was performed to compare differences in metabolite N.I. and concentration between term and preterm-delivered women within the group. *Post hoc* adjustment for multiple comparisons was undertaken by the Bonferroni test. Values are presented as mean ± SEM (except where otherwise stated). The relationships between maternal clinical data, acetate concentration, and ^1^H-NMR metabolite N.I. were determined by Pearson’s correlation coefficients and *P* values <0.05 were considered statistically significant. The predictive capacity of the CVF metabolites for PTB was determined by ROC curves for the following comparisons:

- Preterm (<37 weeks) vs. term births,
- Preterm birth <32 vs. >32 weeks gestation,
- <2 vs. >2 weeks from presentation to delivery.

A cutoff value of acetate concentration for predicting PTB <37 weeks gestation was also calculated from the ROC curve. The combined predictive accuracy of FFN, ultrasound CL, and CVF acetate was estimated by binary logistic regression and combined area under the ROC curve analysis (MedCalc Software bvba, BE, USA).

## Results

### Participants’ Clinical Details and Pregnancy Outcome

Participants’ clinical characteristics and delivery outcomes are summarized in Table 1. Also, 18.3% (15/82) of the women delivered preterm (<37 weeks), with mean duration of time between presentation and delivery of 15.7 ± 3.5 (preterm) and 60.3 ± 3.2 days (term). Of the 15 PTBs recorded, 8 women (9.8% of the total study population) delivered before 32 weeks of gestation.

**Table 1:** **Maternal clinical and demographic characteristics**.

Characteristics	Symptomatic pregnant women 24–36 weeks gestation	
	Preterm (*N* = 15)	Term (*N* = 67)
Age (years)	31.1 ± 2.0^b^ (22–48, *n* = 15)	26.6 ± 0.7 (16–44, *n* = 67)
BMI (kg m^−2^)	28.9 + 1.9^a^ (20.1–42.5, *n* = 11)	25.1 ± 0.6^a^ (17.4–41.6, *n* = 56)
Previous history of PTB (*n*)	5	10
Cigarette smokers, *n* (%)	1(7)	10(15)
Cervical length (mm)	21.1 ± 4.8^a^ (7–45, *n* = 8)	30.8 ± 1.6^ba^ (11–54, *n* = 37)
Fetal fibronectin conc. (ng/ml)	188 ± 85^ba^ (5–501, *n* = 6)	15.2 ± 3^a^ (1–74, *n* = 37)
Gestational age at presentation (days)	206 ± 5.4 (177–237, *n* = 15)	213 ± 3.1 (139–254, *n* = 67)
Gestational age at delivery (days)	221 ± 5.7 (187–257, *n* = 15)	275 ± 1.1 (260–295, *n* = 61)
Vagina pH	4.4 ± 0.3^a^ (3.6–6.1, *n* = 7)	4.2 ± 0.1^a^ (3.6–6.1, *n* = 36)
Prevalence of PTB, %	18.3	

*Data are presented as mean ± SEM (range, *n*); BMI, body mass index; PTB, preterm birth; *N*, total number of term- or preterm-delivered women; *n*, actual number of women for each clinical parameter obtained*.

*^a^Reduced study population (*n*) due to absence of participants’ consent and/or data*.

*^b^Differences between preterm and term-delivered women, P < 0.05*.

### ^1^H-NMR Derived Normalized Integrals

A representative ^1^H-NMR spectrum of identified metabolites is shown in Figure 1.

**Figure 2:**
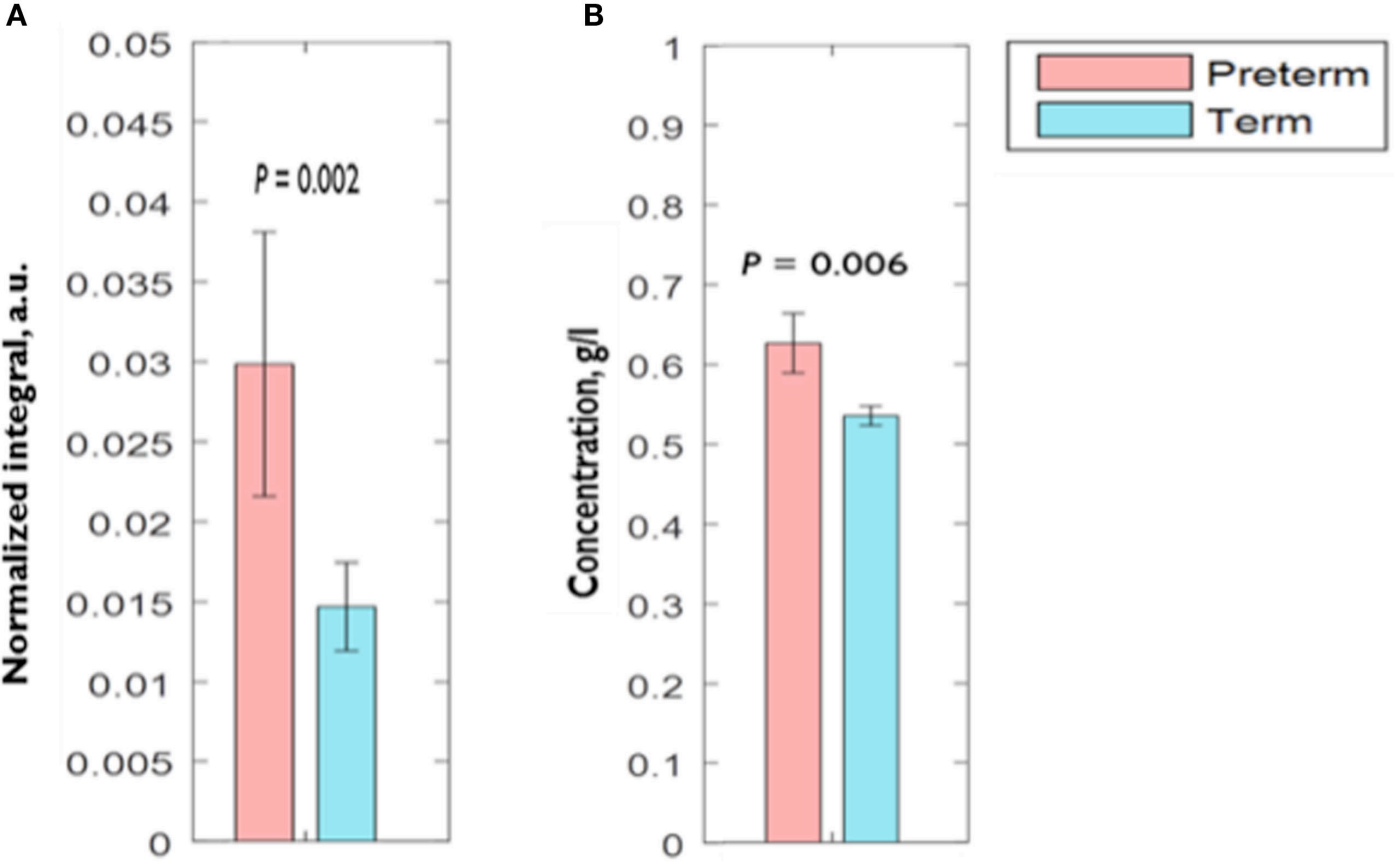
**Comparison of CVF (A) acetate normalized integral measured by ^1^H-NMR (B) acetate concentration measured by spectrophotometry between preterm and term women**. *Error bars* = SEM; *a.u*., arbitrary unit.

Comparison of CVF metabolite N.I.s derived by ^1^H-NMR showed significantly higher (0.03 ± 0.01 vs. 0.01 ± 0.003 a.u.; *P* = 0.002) acetate N.I. in women who delivered preterm compared to their term counterparts. There was about threefold increase in acetate N.I. in women who ultimately delivered preterm (Figure 2A).

**Figure 3:**
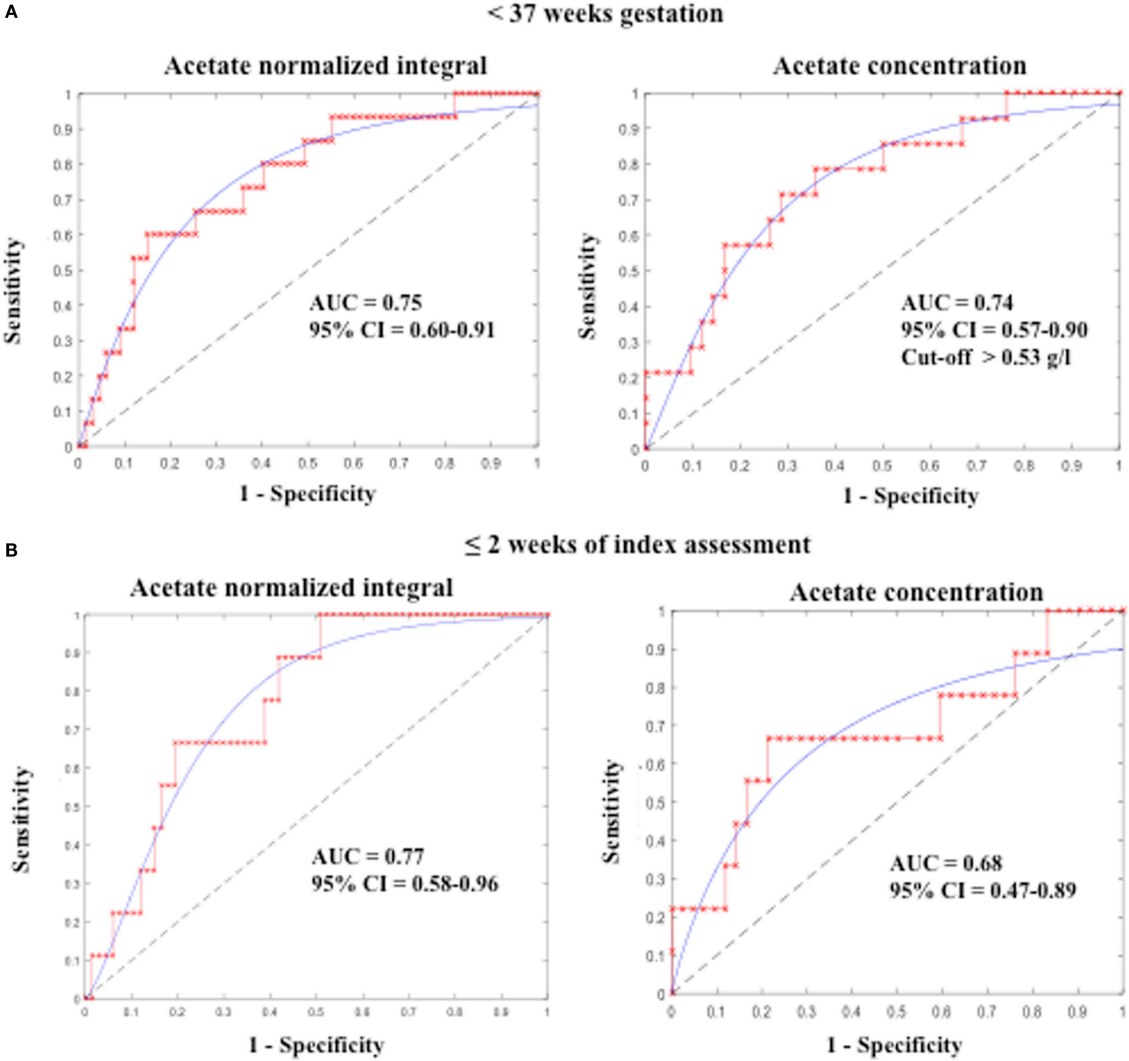
**Predictive accuracy of CVF acetate for preterm birth (A) before 37 weeks and (B) within 2 weeks of index assessment in pregnant women with symptoms of preterm labor**. CVF acetate is measured by ^1^H-NMR (acetate normalized integral) and spectrophotometry (acetate concentration).

Additionally, ROC curve analysis showed that acetate N.I. was predictive of PTB <37 weeks (area under the ROC curve, AUC = 0.75, 95% CI = 0.60–0.91), <32 weeks (AUC = 0.73, 95% CI = 0.53–0.94), and delivery within 2 weeks of the index assessment (AUC = 0.77, 95% CI = 0.58–0.96) (Table 2 and Figure 3).

**Table 2:** **Predictive accuracy of CVF acetate ^1^H-NMR normalized integral and concentration for preterm birth**.

	AUC	95% CI	Sens (%)	Spec (%)	PPV (%)	NPV (%)	LR+	LR−	*P* value
**Delivery <37 weeks gestation**									
Acetate N.I.	0.75	0.60–0.91	60	85	47	91	4.0	0.2	0.001
AceConc	0.74	0.57–0.90	71	71	36	92	2.5	0.4	0.002
**Delivery <32 weeks gestation**									
Acetate N.I.	0.73	0.53–0.94	88	59	21	97	2.1	0.5	0.01
AceConc	0.63	0.40–0.87	72	63	19	95	2.0	0.5	0.13
**Delivery within 2 weeks of assay**									
Acetate N.I.	0.77	0.58–0.96	100	49	21	100	2.0	0.5	0.003
AceConc	0.68	0.47–0.89	67	79	29	95	3.1	0.3	0.045

*Acetate N.I., ^1^H-NMR acetate normalized integral; AceConc, acetate concentration measured by spectrophotometric technique; AUC, area under the ROC curve; CI, 95% confidence interval; Sens, sensitivity; Spec, specificity; PPV, positive predictive value; NPV, negative predictive value; LR+, positive likelihood ratio; LR−, negative likelihood ratio; P, significance level*.

Normalized integrals of succinate, formate, lactate, glucose, glutamine/glutamate, alanine, and branched chain amino acids did not differ between term- and preterm-delivered women.

### Acetate Concentration Measured by Spectrophotometry

**Figure 4:**
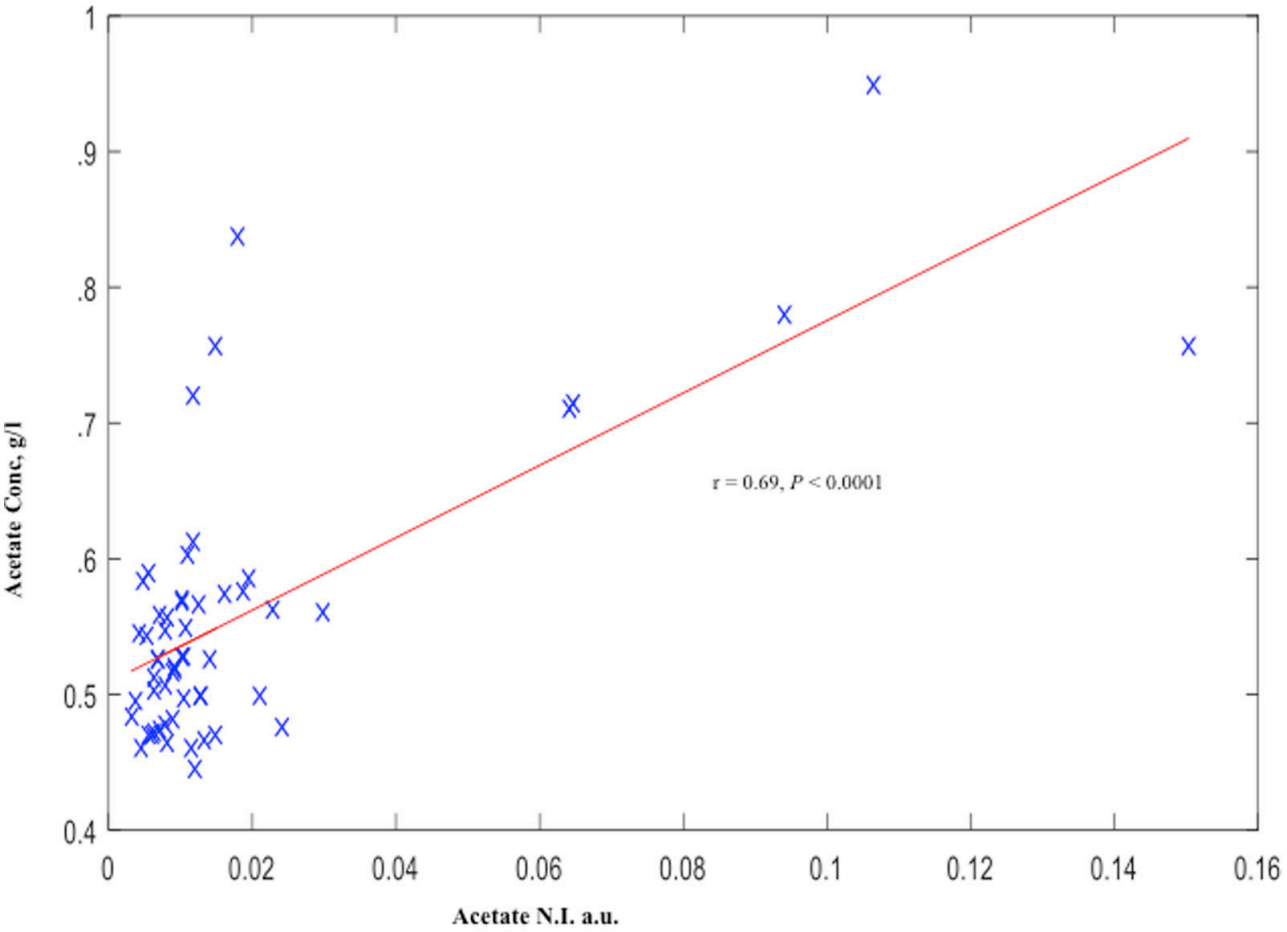
**Cervicovaginal fluid acetate of symptomatic pregnant women estimated by ^1^H-NMR and Spectrophotometry**. *a.u*., arbitrary unit.

Similar to our observation with acetate N.I., AceConc measured by the spectrophotometric technique was significantly higher in the preterm-delivered vs. term-delivered women (0.63 ± 0.04 vs. 0.54 ± 0.01 g/l; *P* = 0.006) (Figure 2B), and correlated strongly with the ^1^H-NMR acetate N.I. (*r* = 0.69, *P* < 0.00001) (Figure 4).

Furthermore, acetate concentration measured by the spectrophotometric technique was predictive of PTB <37 weeks (AUC = 0.74, 95% CI = 0.57–0.90) and delivery within 2 weeks of the index assessment (AUC = 0.68, 95% CI = 0.47–0.89), with an optimal cutoff value of >0.53 g/l. However, its predictive performance for PTB <32 weeks did not attain statistical significant in this limited sample (Table 2; Figure 3).

### CVF FFN, Ultrasound CL, and Vaginal pH

The women destined to deliver preterm had significantly shorter mean CL (21.1 ± 4.8 vs. 30.8 ± 1.6 mm) and greater than 12-fold higher FFN concentration (188 ± 85 vs. 15.2 ± 3 ng/ml) compared to their term counterparts (Table 1 and Figure S3 in Supplementary Material). Both FFN (*r* = −0.7, *P* < 0.00001) and CL (*r* = 0.4, *P* = 0.01) correlated with gestational age at delivery (GAAD) (an indication of term or PTB) (Figure S4 in Supplementary Material).

Also, analysis of the area under the ROC curve showed FFN, and ultrasound CL were predictive of PTB <37 weeks (Table 3).

**Table 3:** **Individual and combined predictive values of CVF acetate, fetal fibronectin, and cervical length for preterm birth**.

Test	AUROC (95% CI)
Acetate N.I.	0.75 (0.60–91)
AceConc	0.74 (0.57–0.90)
Fetal fibronectin	0.76 (0.53–1.0)
Cervical length	0.73 (0.52–0.94)
Fetal fibronectin + cervical length	0.84 (0.64–1.0)
Fetal fibronectin + cervical length + acetate N.I.	0.86 (0.69–0.95)
Fetal fibronectin + cervical length + AceConc	0.81 (0.62–0.93)

*AUROC, area under the ROC curve; Acetate N.I., ^1^H-NMR acetate normalized integral; AceConc, acetate concentration measured by spectrophotometric technique*.

Vaginal pH did not differ between term- and preterm-delivered women (Table 1), but it correlated with acetate (*r* = 0.4, *P* = 0.01) and lactate N.I.’s (*r* = −0.6, *P* < 0.00001) (Figure S5 in Supplementary Material).

### Combined Predictive Performance of CVF Acetate, FFN, and Ultrasound CL

**Figure 5:**
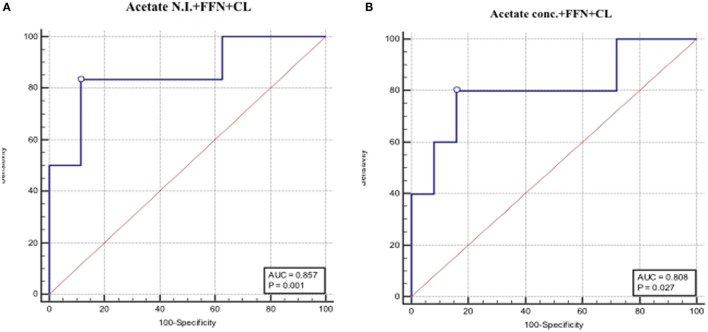
**AUROCs depicting combined PTB predictive performance of fetal fibronectin (FFN), cervical length and CVF acetate estimated by ^1^H-NMR (A), and spectrophotometry (B)**.

Apart from the observation of comparable predictive capacities between CVF acetate, FFN, and ultrasound CL (Table 3), combining CVF acetate estimated by ^1^H-NMR and spectrophotometry with these widely used clinical assessment markers improved the prediction of PTB – the inclusion of either CVF acetate N.I. or acetate concentration with FFN and ultrasound CL in a binary logistic regression model improved prediction of PTB before 37 weeks gestation (AUC = 0.86, sensitivity 83%, specificity 89%, LR+ 7.3, and LR− 0.2 for acetate N.I. and AUC = 0.80, sensitivity 80%, specificity 84%, LR+ 5.0 and LR− 0.2 for acetate concentration) than each of the three markers considered singly (Figures 5A,B). However, the combination of FFN and ultrasound CL alone indicated comparable predictive power to all three markers combined together (AUC = 0.84) (Table 3).

## Discussion

We have employed ^1^H-NMR spectroscopy to characterize the CVF metabolite profiles of a cohort of pregnant women presenting with symptoms suggestive of threatened PTL at mid-gestation and determined their prognostic accuracy for PTB. Consistent with our previous report (18), we confirmed that CVF acetate appears predictive of PTB in this cohort. Furthermore, we have now validated this observation in this report employing commercially available enzyme-based spectrophotometric assay kits for acetic acid. We observed that CVF acetate predicted PTB as well as quantitative FFN and ultrasound CL, and that when combined with these determinations, predictive accuracy for preterm delivery before 37 weeks gestation improved moderately. To our knowledge, this comparative analysis is the first of its kind.

Of the metabolites identified in the ^1^H-NMR spectra of CVF samples of women presenting with features of PTL, only acetate levels were associated with preterm delivery. The women destined to deliver prematurely had a twofold higher acetate N.I. compared to those who delivered at term. Acetate N.I. was also prognostic of PTB before 37 and 32 weeks of gestation, as well as of delivery within 2 weeks of the index assessment. Measurement of CVF acetate levels by ^1^H-NMR, which is a marker of altered vaginal microbiota (6), is associated with imminent PTB.

However, acetate N.I. determination by ^1^H-NMR assessment does not lend itself to clinical use as an assay technique. We therefore validated ^1^H-NMR data by determining absolute concentrations of acetate in CVF by employing a commercial spectrophotometric assay technique from a randomly selected subset of the same symptomatic pregnant women. We determined that acetate concentration derived by spectrophotometry correlated strongly with ^1^H-NMR-derived acetate N.I. being significantly higher in the women who ultimately delivered preterm. Additionally, acetate concentration was predictive of preterm delivery before 37 weeks gestation and of delivery within 2 weeks of the index assessment. However, predictive potential for delivery before 32 weeks did not attain statistical significance as only eight women in this subset delivered before 32 weeks gestation.

Conditions characterized by altered vaginal microflora such as intermediate flora, aerobic vaginitis, and BV have been implicated in infection/inflammation-induced preterm delivery. However, BV is the most investigated (3). The earlier the gestational age at the onset of PTL and possibly preterm premature rupture of membranes leading to PTB, the higher the likelihood of genital tract infection and inflammation (e.g., chorioamnionitis) (20). More than half of the women who delivered preterm in this study and approximately 10% of the entire study population delivered very prematurely before 32 weeks. In this category of PTBs, subclinical intrauterine infection is most often evident (20). As demonstrated in this study, identification of metabolic by-products (such as acetate) of pathogenic anaerobic bacteria seen in the above conditions could be used as a non-invasive, quick, and cost-effective proxy marker for the characterization of the prevailing vaginal microbial community and the attendant inflammatory state. Understanding these metabolite patterns in pregnant women may, therefore, aid understanding of the mechanisms of inflammation-induced preterm delivery, as well as uncover potential novel therapies.

We also demonstrated a relationship between vaginal pH, acetate, and lactate N.I.’s (Figure S5 in Supplementary Material), consistent with a potential metabolite signature pattern in women destined to deliver preterm. Vaginal pH increases with elevated acetate production by anaerobic bacteria (e.g., *Gardnerella, Prevotella, Bacteroides, Mobilincus, Mycoplasma*, etc.) characteristic of BV, while pH decreases with increase in lactate predominantly produced by *Lactobacillus* species characteristic of a healthy vaginal microbiota (6). These changes, together with increased recruitment of neutrophils, pro-inflammatory cytokines, and chemokines (e.g., IL-6 and IL-8), are also strongly associated with early third trimester rupture of fetal membranes (8, 21) often a precursor of idiopathic spontaneous PTB.

Although quantitative FFN and ultrasound CL are the most utilized clinical assessment markers for distinguishing women at risk of PTB in asymptomatic and symptomatic cohorts, there use is associated with a high rate of false positive results, low sensitivity, and positive predictive values for PTB (2). Combining both tests in midtrimester appears to improve their predictive accuracy of PTB (22). Furthermore, an association between genital tract infection/inflammation, short cervix, membrane activation and disruption, and leakage of FFN has been reported (8, 23). In this cohort, CVF acetate appeared as predictive of PTB as CL and FFN separately. Considering the limitations of FFN and ultrasound CL, we combined them with CVF acetate to determine whether the prognostication of PTB could be improved by the multiple biomarkers. We have observed that the combination yielded improved prediction of PTB.

In summary, we have reported that elevated CVF acetate (produced by mixed anaerobes) in women with symptoms of PTL appears predictive of preterm delivery, as well as delivery within 2 weeks of presentation, independently. This is supported by the established association of vaginal microbiota dominated by mixed anaerobic bacteria with the initiation of inflammation-associated PTL and birth. In pregnant women presenting with symptoms suggestive of PTL, a clinical assay of acetate in CVF may prove of clinical utility for predicting PTB singly and in combination with quantitative FFN and ultrasound CL, if our observations are confirmed in larger studies. Whether CVF acetate has clinical utility for predicting PTB in asymptomatic pregnant women is yet to be determined.

## Author Contributions

EA conducted the laboratory experiments, analyzed and interpreted the data, and drafted the manuscript. SR supervised the NMR experiments and contributed to data analysis and the manuscript. VS recruited study participants, obtained tissue samples, carried out the clinical studies, and contributed to data analysis and the manuscript. GS contributed to the study concept, project supervision, and the manuscript. MP supervised NMR experiments and contributed to the manuscript. DA conceived the studies, supervised the project, contributed to the analysis and interpretation of data, and writing the manuscript. All authors approved the final draft for publication. Parts of this work have been presented at the 63rd Annual Scientific Meeting of the Society for Reproductive Investigation, Montreal 2016, O-090 (Oral); the 18th annual conference of the British Maternal and Fetal Medicine Society, Birmingham 2016, PP.16 (Poster); and the 24th annual meeting and exhibition of the International Society of Magnetic Resonance in Medicine, Singapore 2016, 2414 (Poster).

## Conflict of Interest Statement

The authors declare that this research was conducted in the absence of any commercial or financial relationships that could be construed as a potential conflict of interest.

## Supplementary Material

### Acetic Acid Spectrophotometric Assay

The assay principle is based on the determination of acetate concentration by the generation of NADH from NAD+, which is then quantified by the increase in absorbance at 340 nm (24). The enzymes involved in the four-step reaction and supplied with the assay kit include: (1) acetate kinase, (2) phosphotransacetylase, (3) ADP-glucokinase (ADP-GK), and (4) glucose-6-phosphate dehydrogenase (G6P-DH). Acetate is converted through the following reaction, which is positively related to the eventual formation of NADH:

1. Acetic acid + ATP  acetyl-phosphate + ADP
2. Acetyl-phosphate + CoA  acetyl-CoA + P_i_
3. ADP + D-glucose  glucose-6-phosphate + AMP
4. Glucose-6-phosphate + NAD^+^  6-phosphogluconate + NADH + H^+^

The reaction is a positive reaction indicated by the increase in absorbance. The acetic acid GK assay kit is an endpoint type assay specific for acetate with its lowest sensitivity limit ~0.1 g/l (*R*^2^ = 0.9997). A calibration curve indicating the linearity of K-ACETGK 08/14 was produced by plotting absorbance at 340 nm against concentrations of the acetic acid standards (gram per liter) (*R*^2^ = 0.975, detection limit: 1.8 g/l) (Figure S2 in Supplementary Material). Different concentrations (calibrators) of the acetic acid standard (1.8 g/l) were made be serial dilution (i.e., 1.8, 1.2, 0.9, 0.6, 0.45, 0.36, 0.3, 0.225, 0.2, and 0.15 g/l). From the calibration curve, concentrations of acetate in the samples were ascertained after measurement of their absorbance at 340 nm.

Figure S1 **2-D ^1^H-^13^C HSQC NMR spectrum of cervicovaginal fluid showing confirmed metabolites at 294 K**. *BCAA*, branched chain amino acids (leucine, isoleucine, and valine); *HSQC*, heteronuclear single quantum correlation spectroscopy.

Figure S2 **Calibration curve indicating the linearity of K-ACETGK**. The reactions from which this calibration curve was generated were performed at 37°C for about 5 min using a TECAN Infinite M200 microplate reader (detection limit = 1.8 g/l or 0.03 mol/l).

Figure S3 **Clinical assessment methods for preterm birth (A) quantitative fetal fibronectin level and (B) ultrasound cervical length in relation to delivery outcomes**. Data are presented as mean ± SE. **P* value < 0.05.

Figure S4 **Association of quantitative fetal fibronectin (FFN) level (A), ultrasound cervical length (B), and gestational age at delivery (GAAD)**.

Figure S5 **Association of vaginal pH and CVF acetate (A) and lactate (B) normalized integrals in symptomatic pregnant women**. *a.u*., arbitrary unit.
