# Supplementary figures and images for: Cervicovaginal Fluid Acetate: A Metabolite Marker of Preterm Birth in Symptomatic Pregnant Women
Source: Front Med (Lausanne). 2016 Oct 10;3:48. doi: 10.3389/fmed.2016.00048 (PMC5056530; doi:10.3389/fmed.2016.00048)

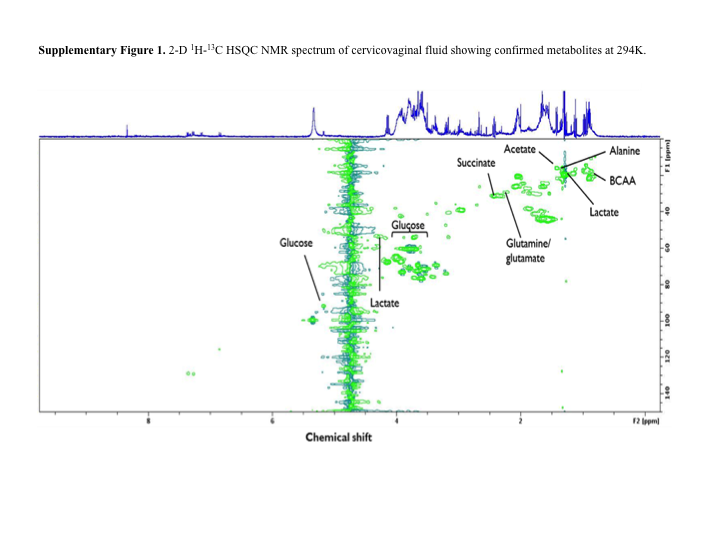

Supplement: Figure S1 — 2-D 1H-13C HSQC NMR spectrum of cervicovaginal fluid showing confirmed metabolites at 294 K. BCAA, branched chain amino acids (leucine, isoleucine, and valine); HSQC, heteronuclear single quantum correlation spectroscopy. [file image_1.tiff]

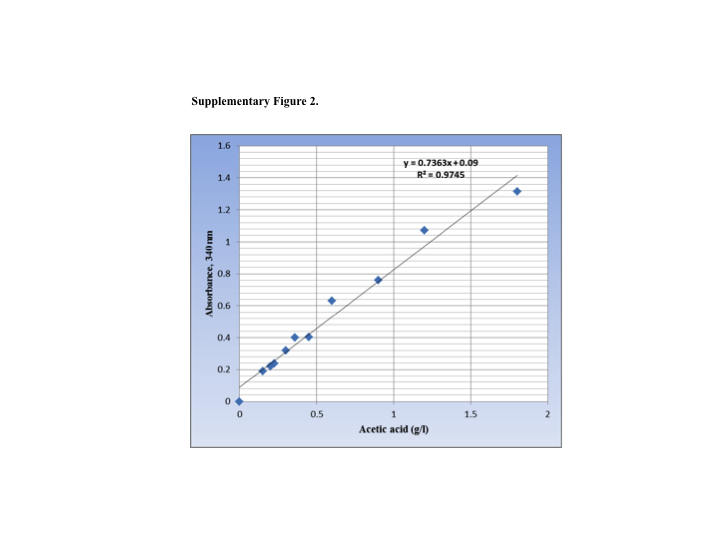

Supplement: Figure S2 — Calibration curve indicating the linearity of K-ACETGK. The reactions from which this calibration curve was generated were performed at 37°C for about 5 min using a TECAN Infinite M200 microplate reader (detection limit = 1.8 g/l or 0.03 mol/l). [file image_2.tiff]

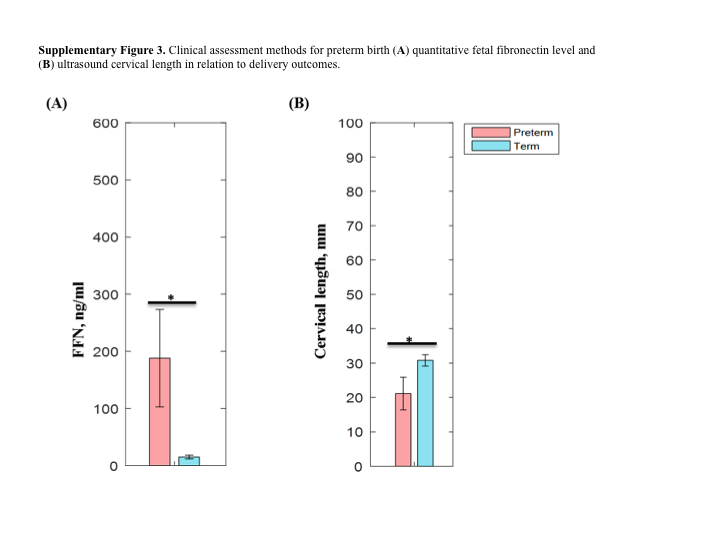

Supplement: Figure S3 — Clinical assessment methods for preterm birth (A) quantitative fetal fibronectin level and (B) ultrasound cervical length in relation to delivery outcomes. Data are presented as mean ± SE. *P value < 0.05. [file image_3.tiff]

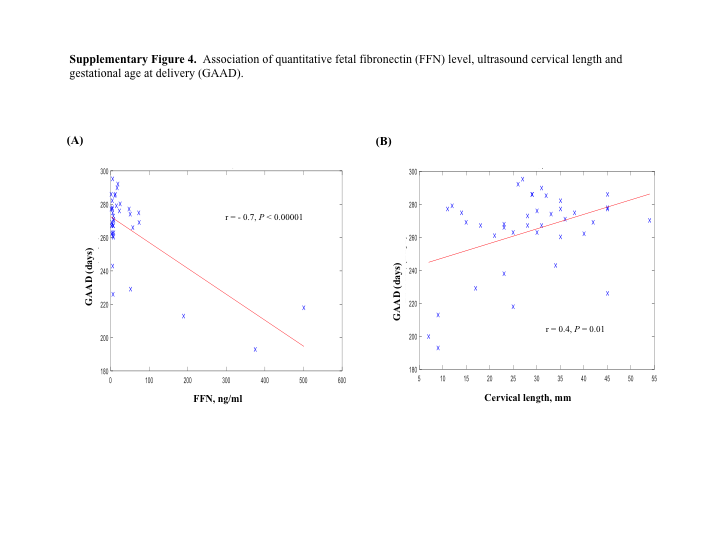

Supplement: Figure S4 — Association of quantitative fetal fibronectin (FFN) level (A), ultrasound cervical length (B), and gestational age at delivery (GAAD). [file image_4.tiff]

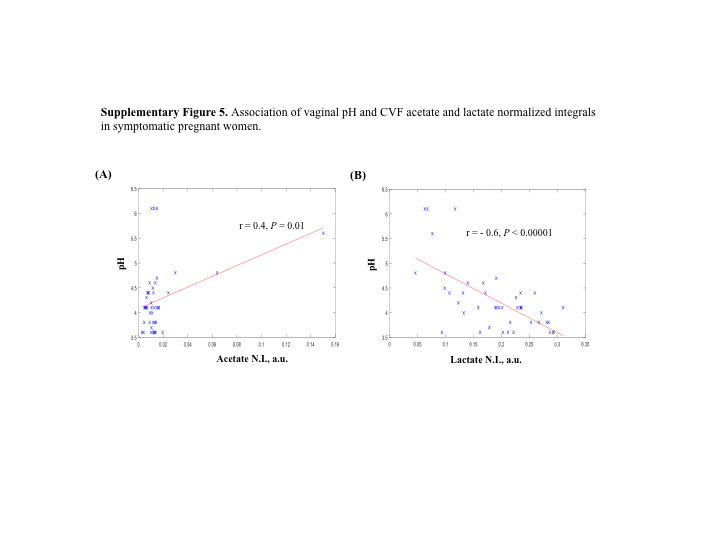

Supplement: Figure S5 — Association of vaginal pH and CVF acetate (A) and lactate (B) normalized integrals in symptomatic pregnant women. a.u., arbitrary unit. [file image_5.tiff]
